# Real-world experience of galcanezumab in the prevention of migraine in Spain: a systematic literature review

**DOI:** 10.3389/fneur.2024.1502475

**Published:** 2024-11-21

**Authors:** Patricia Pozo-Rosich, David García-Azorín, Silvia Díaz-Cerezo, Julia Fernández-Montoya, Héctor David de Paz, Mercedes Núñez

**Affiliations:** ^1^Neurology Department, Hospital Universitari Vall d’Hebron, Barcelona, Spain; ^2^Department of Medicine, Universitat Autònoma de Barcelona, Barcelona, Spain; ^3^Neurology Department, Hospital Universitario Río Hortega, Valladolid, Spain; ^4^Department of Medicine, Faculty of Medicine, Universidad de Valladolid, Valladolid, Spain; ^5^Medical Department, Lilly Spain, Alcobendas, Spain; ^6^Health Outcomes Research Department, Outcomes’10 S.L., Castellón de la Plana, Spain

**Keywords:** CGRP, calcitonin gene-related peptide-targeting therapies, migraine, monoclonal antibodies, observational studies, prophylaxis, systematic review

## Abstract

**Introduction:**

In the context of migraine preventive therapy, new therapeutic modalities such as monoclonal antibodies targeting the calcitonin gene-related peptide receptor (CGRP) or ligand offer potential advantages over traditional preventive treatments.

**Methods:**

This systematic literature review gathered recent real-world evidence from Spain on the use of galcanezumab, a CGRP-targeting treatment, in migraine patients. The review included observational studies in English or Spanish, published from August 2020 to August 2023, following the Preferred Reporting Items for Systematic Reviews and Meta-Analyses (PRISMA) and Cochrane guidelines.

**Results:**

A total of 29 publications involving 2,592 Spanish adult patients were identified, reporting relevant information on clinical outcomes (treatment effectiveness and safety), treatment persistence and patterns (switching from other therapies and time to discontinuation and restart), and patient-reported outcomes (including satisfaction with treatment). The most frequently reported variables were related to the clinical effectiveness of galcanezumab, demonstrating a significant reduction in monthly migraine days and monthly headache days. Additionally, adverse impact of headache per HIT-6 (Headache Impact Test-6) and disability per MIDAS (Migraine Disability Assessment) also improve. Studies also showed that up to 12-month persistence to galcanezumab ranged from 76.8 to 59.8%. Serious adverse events were rare. None of the publications included health-related quality of life data, either generic or migraine-specific. One study highlighted that galcanezumab treatment would offer high patient satisfaction for people with migraine.

**Conclusion:**

The real-world evidence on the use of galcanezumab treatment among the Spanish population shows that its effectiveness, persistence, safety, and impact on health burden align with findings from clinical trials and observational studies conducted in other countries. Future studies should incorporate health-related quality of life data to gain a more holistic understanding of this treatment’s impact.

## 1 Introduction

Migraine is a neurological disorder characterised by recurrent headache attacks of moderate-to-severe pain intensity lasting 4 to 72 h when untreated. It is often accompanied by increased sensitivity to light, noise, and odours or gastrointestinal disorders such as nausea or vomiting (1).

Migraine affects over 1 billion people worldwide, with a higher prevalence in women (3:1 ratio to men), often beginning at puberty and mostly affecting those aged 35–39 years old (2). Depending on the monthly frequency of the attacks, episodic migraine (EM) is defined as a headache occurring <15 days per month, while chronic migraine (CM) is defined as a headache occurring ≥15 days per month, of which at least 8 days meet the criteria for a migraine attack and/or respond to acute migraine-specific medication (3, 4).

Migraine therapy can be acute and/or preventive treatments. The acute therapy restores patient’s functional status by aborting the headache stage and associated symptoms of migraine (5). On the other hand, the preventive therapy aims to decrease the frequency, severity, and duration of migraine attacks (6–8). The decision to start with preventive treatment must be individualised, and is indicated when the attacks are frequent and disabling, the patients’ quality of life is impaired or when acute treatments are not sufficiently effective or not well tolerated (5). In this context, the insufficient efficacy and tolerability of traditional treatments for migraine has prompted the search for new therapeutic strategies for the preventive treatment of migraine such as monoclonal antibodies (mAb) against the calcitonin gene-related peptide (CGRP) receptor (erenumab) or against CGRP ligand (eptinezumab, fremanezumab and galcanezumab) or gepants (9–16). The recent commercialization by the Spanish authorities of anti-CGRP therapies, including galcanezumab, has allowing many investigators and industry to collect data in real-world settings.

The latest update of the European Headache Federation (EHF) guidelines as well as the American Headache Society position statement update suggest using mAb against the CGRP pathway and gepants as a first line treatment option for those patients who require a preventive treatment (15, 17). In Spain, preventive treatments such as anti-CGRP mAbs are recommended in patients who suffer ≥4 migraine attacks per month and previous treatment failures (18, 19).

Galcanezumab was authorised in the European Union in November 2018 indicated for the prophylaxis of migraine in adults who have at least 4 migraine days per month (20). However, the Spanish National Health System (NHS) decided full reimbursement would be for those patients with >8 or more monthly migraine days (MMDs) and > 3 or more failures of previous treatments used with sufficient doses for at least 3 months (including botulin toxin; onabotulinumtoxin A [OnabotA] in patients with CM) (21).

Even though there is a growing body of real-world data examining galcanezumab’s effectiveness and safety in migraine prevention, a comprehensive analysis of its utilisation within the Spanish population remains a notable gap in the literature. The purpose of this systematic literature review is to provide an in-depth analysis of the available evidence regarding galcanezumab’s use for migraine prevention, including effectiveness, safety, treatment patterns, and patient-reported outcomes. Through this review, we seek to facilitate evidence-based decision-making and enhance understanding of galcanezumab’s potential role in preventing the burden of migraine within the Spanish adult population.

## 2 Materials and methods

A systematic review of the literature was conducted following the recommendations included in the Preferred Reporting Items for Systematic Reviews and Meta-Analyses (PRISMA) and Cochrane guidelines (22, 23).

### 2.1 Search strategy

A search was conducted in the international databases PubMed/Medline and Cochrane library, and the Spanish databases *Medicina en Español* (MEDES) and *Índice Bibliográfico Español en Ciencias de la Salud* (IBECS) using standardised search filters and terms.

The search was conducted using free-text and MeSH (Medical Subject Headings) terms for PubMed/MedLine database, both combined with the Boolean connectors “OR” and “AND.” Details of the search strategy and terms used can be found in Supplementary Table S1.

Additional searches in grey literature (Google and Google Scholar) and the European and American medical societies (European Academy of Neurology [EAN], American Academy of Neurology [ANN], Spanish Society of Neurology [SEN], and EHF) were carried out to identify studies published in Spain. The bibliographic references of the selected articles were also reviewed to search for relevant publications that might have not been detected in the literature search.

### 2.2 Eligibility criteria

The systematic review included data from observational studies involving Spanish adult patients with migraine treated with galcanezumab. These studies, published in English or Spanish between August 2020 and August 2023, reported data regarding the patient characteristics (subpopulation of interest), clinical outcomes (effectiveness, safety), treatment persistence and patterns (switch from other therapies and time to discontinuation and re-initiation), and patient reported outcomes (PROs) indicating, among others, satisfaction with the treatment. Additionally, relevant conference abstracts published between August 2021 and August 2023 were also included. Full details of the inclusion and exclusion criteria can be found in Supplementary Table S2.

### 2.3 Study selection and data extraction

Two independent reviewers screened all identified publications in accordance with PRISMA recommendations (23) and extracted all data. Discrepancies were resolved by consensus. All screening was recorded using the developed inclusion criteria as described above. Reviewers were not blinded to any study information. A standardised data extraction form was used to analyse and extract the data from the selected studies.

Data extracted included sociodemographic and clinical characteristics, clinical outcomes, treatment persistence and adherence, treatment patterns and PROs.

The reporting quality of included studies was assessed using the Strengthening the Reporting of Observational Studies in Epidemiology (STROBE) statement (24). Study quality was assessed by one reviewer and verified by a second reviewer, with discrepancies being resolved by consensus or involvement of a third team member. The quality assessment was conducted only for the full-text articles (not the conference abstracts).

### 2.4 Ethical approval

This article is based on previously conducted studies and does not contain any new studies with human participants or animals performed by any of the authors.

## 3 Results

### 3.1 Selected studies

**Figure 1:**
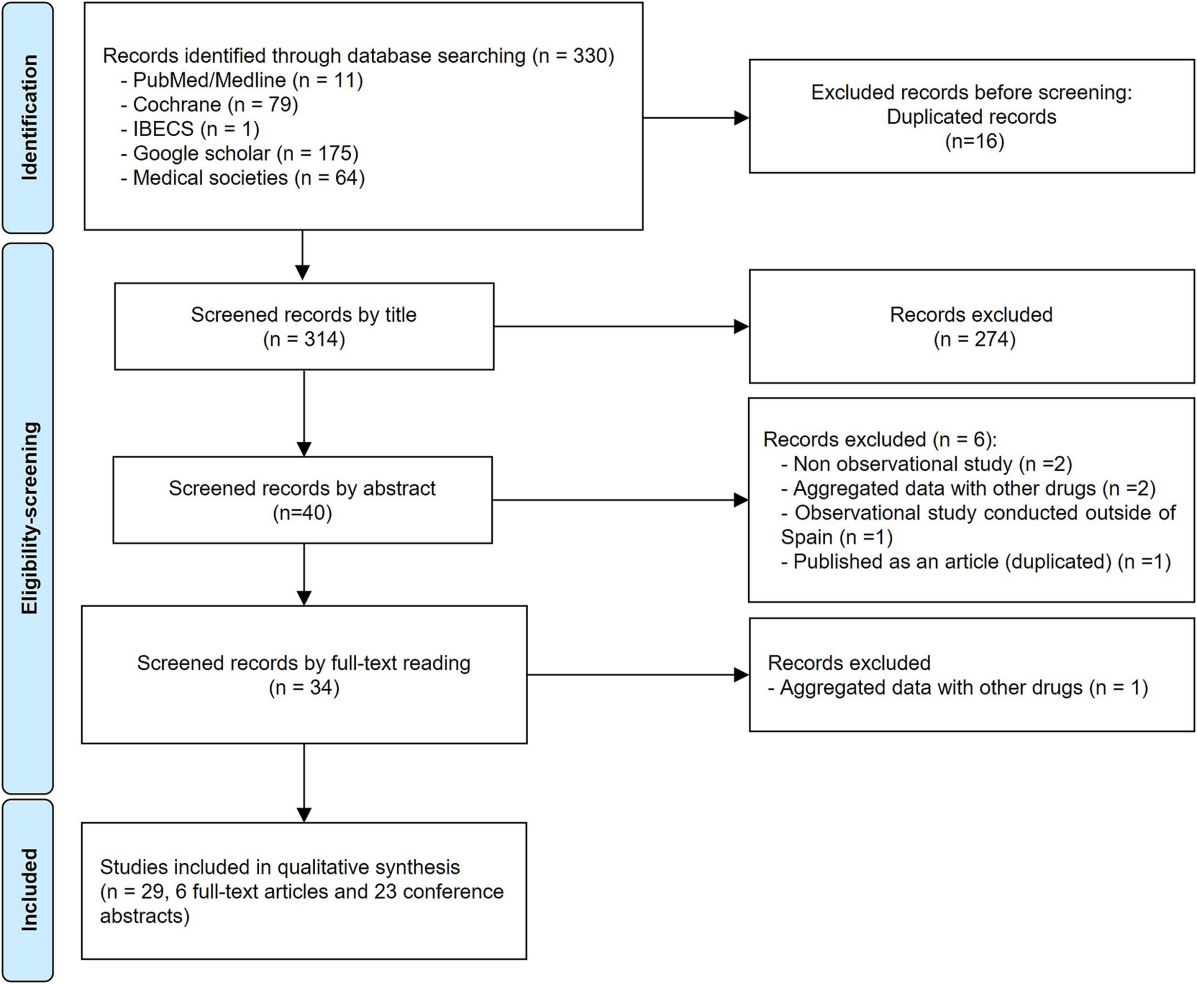
PRISMA diagram showing the study selection progress.

The search in the databases and grey literature yielded 330 records. Out of these, 29 publications (6 full-text articles and 23 conference abstracts) were included and analysed for the present systematic review (Figure 1).

Approximately half of the included publications presented a prospective (*n* = 13, 44.8%) or retrospective (*n* = 15, 51.7%) design, while one had a cross-sectional (*n* = 1, 3.4%) design (Tables 1, 2). Overall, a total population of 2,592 Spanish patients with migraine in galcanezumab treatment were included in all reviewed studies (the different publications related to a same study were grouped).

**Table 1:** Main characteristics of selected publications/studies reporting galcanezumab data only.

Author (year)	Region	Study design	Inclusion criteria	Population size	Endpoints				
					Clinical outcomes		Treatment persistence and adherence	Treatment patterns	PROs
					Effectiveness	Safety			
Lopez-Bravo (2022) (31)	*Navarra*	Single-centre Cross-sectional Longitudinal study	Diagnosis according to ICHD criteria with ≥8 MHDs and galcanezumab treatment	30	MMDs MHDs HIT-6 MIDAS	X	X	X	Treatment satisfaction (TSQM-1.4) Anxiety and depression (HADS-A, HADS-D)
Patier (2022) (28)	*Madrid*	Retrospective Single-centre Longitudinal descriptive study	Patients treated with erenumab and switched to galcanezumab with at least 24-week follow-up from therapy initiation	15	MMDs Acute medication use Response rate	X	X	X	
Fabregat Fabra (2022) (32) [ABSTRACT] (Galca-only Consortium)	Spain	Multicentre Prospective Longitudinal and cohort descriptive study	People with migraine treated with galcanezumab	1,004	MHDs HIT-6		X	X	PGI
Fernández Fernández (2021) (33) [ABSTRACT]	*Catalonia*	Single-centre, Prospective Longitudinal descriptive study	Patients with CM having clinical indication for galcanezumab treatment (3 failed preventives)	73	MHDs	X		X	Global improvement reported by patient
Fernández Fernández (2022) (52) [ABSTRACT] (Galca-only Consortium)	Spain	Multicentre Retrospective Cohort descriptive study	People with migraine treated with galcanezumab	1,004	MHD HIT-6	X	X		
Fernández Soberón (2022) (53) [ABSTRACT]	*Basque Country*	Single-centre Retrospective Cohort descriptive study	People with migraine treated with galcanezumab	52	MMDs MIDAS HIT-6	X	X	X	
Membrilla-López (2021) (71) [ABSTRACT]	*Madrid*	Single-centre Prospective Cohort comparative study	People with migraine with MOH and treated with galcanezumab	54	MMDs MIDAS HIT-6			X	
Membrilla-López (2022) A (72) [ABSTRACT]	*Madrid*	Single-centre Prospective Longitudinal comparative study	People with migraine with comorbid MOH and treated with galcanezumab	46	MHDs HIT-6 MIDAS		X	X	
Membrilla-López (2022) B (73) [ABSTRACT]									
Mínguez-Olaondo (2022) (34) [ABSTRACT] (Galca-only Consortium)	Spain	Multicentre Retrospective Longitudinal and cohort comparative study	People with migraine treated with galcanezumab with and without OnabotA	787 patients: OnabotA: 205 nonOnabotA: 582	MMDs MHDs HIT-6 MIDAS			X	PGI
Núñez Lozano (2022) (27) [ABSTRACT] (ORYGAM study)	Spain	Multicentre Retrospective Longitudinal descriptive study	People with migraine treated with galcanezumab	314			X	X	
Obach Baurier (2022) A (54) [ABSTRACT] (Galca-only Consortium)	Spain	Multicentre Retrospective Longitudinal descriptive study	Patients treated with galcanezumab for high frequency (>7 attacks/month, refractory to 3 oral preventive treatments) or CM patients refractory to OnabotA.	1,004	MHDs HIT-6		X		
Obach Baurier (2022) B (74) [ABSTRACT] (Galca-only Consortium)									
Vargas Mendoza (2022) (55) [ABSTRACT]	*Asturias*	Single-centre Prospective Cohort comparative study	People with migraine treated with galcanezumab	41	MHDs HIT-6				
Zunzunegui Arroyo (2022) (56) [ABSTRACT]	*Asturias*	Single-centre Prospective Cohort comparative study	People with migraine treated with galcanezumab	80	MMD				

ICHD-3, International Classification of Headache Disorders criteria; CM, chronic migraine; MMDs, monthly migraine days; MHDs, monthly headache days; HIT-6, Headache Impact Test-6; MIDAS, Migraine Disability Assessment; PROs, patient-reported outcomes; TSQM-1.4, Treatment Satisfaction Questionnaire for Medication version 1.4; HADS, Hospital Anxiety (A) and Depression (D) Scale; VAS, Visual Analogue Scale; PGI, Patient Global Impressions scale; OnabotA, Onabotulinumtoxin A; MOH, Medication overuse headache; MMDs, monthly migraine days.

**Table 2:** Main characteristics of selected publications/studies reporting disaggregated data from different anti-CGRPs treatments.

Author (year)	Region	Study design	Inclusion criteria	Population size	Endpoints				
					Clinical outcomes		Treatment persistence and adherence	Treatment patterns	PROs
					Effectiveness	Safety			
Castaño-Amores (2022) (29)	*Spain*	Multicentre Retrospective Cohort descriptive study	Patients diagnosed with CM or HFEM, treated with a CGRP mAb therapy (erenumab, galcanezumab or fremanezumab) for at least 3 months	286 patients: Erenumab: 86 Galcanezumab: 25 Fremanezumab: 16	MMDs	X	X	X	Subjective improvement in intensity of migraines
López-Moreno (2022) (30)	*Andalusia*	Single-centre Retrospective Cohort descriptive study	Patients diagnosed with HFEM and CM, treated with anti-CGRP mAbs, directly switched to a second anti-CGRP mAb due to inefficacy of the first	14 patients: Erenumab to fremanezumab: 1 Erenumab to galcanezumab: 9 Galcanezumab to erenumab: 4	MMDs MHDs VAS HIT-6 MIDAS	X	X	X	
Muñoz-Vendrell (2023) (25)	Spain	Multicentre Retrospective Cohort comparative study	> 65 years old patients with migraine, according to ICHD-3 and > 8 MMDs and previous history of treatment failure to ≥3 preventive treatments, being OnabotA one of them in CM patients	162 patients: Erenumab: 38 Galcanezumab: 85 Fremanezumab: 39	MHD rMHD	X		X	
Viudez-Martínez (2022) (26)	*Valencia*	Single-centre Retrospective Longitudinal and cohort non-comparative study	Patients diagnosed with CM or EM aged >18 to 65 years who had failed to ≥3 oral preventive medication classes supported by clinical practice guidelines due to lack of efficacy or intolerable side effects. For CM patients, a failure or partial response to current treatment with OnabotA was also requested	142 patients: Erenumab: 83 Galcanezumab: 59	CM MHDs AMSMD HIT-6 MIDAS VAS	X	X	X	
Diaz-Insa (2021) (75) [ABSTRACT]	*Valencia*	Single-centre Prospective Longitudinal and cohort descriptive study	Patients with migraine treated with anti-CGRP mAbs (erenumab and galcanezumab) during ≥3 months.	220 patients: Erenumab: 111 Galcanezumab: 109	MHD	X			
Díaz-Insa (2022-A) (76) [ABSTRACT]	*Valencia*	Single-centre Prospective Longitudinal and cohort descriptive, study	Patients with migraine treated with anti-CGRP mAbs	336 patients: Erenumab: 140 Galcanezumab: 139 Fremanezumab: 57	MMD				
Diaz-Insa (2022-B) (77) [ABSTRACT]	*Valencia*	Single-centre Prospective Cohort descriptive study	Patients with CM	220 patients: Erenumab: 111 Galcanezumab: 109	MMD				
Gracia Moya (2022) (78) [ABSTRACT]	*Catalonia*	Multicentre Retrospective Cohort descriptive study	Patients treated with anti-CGRP mAbs (≥8 MMDs and ≥ 3 previous treatment failures)	339 patients: Erenumab: 182 Galcanezumab: 157	MMDs HIT-6 Response rate	X			
Lamas-Pérez (2021) (79) [ABSTRACT]	*Andalusia*	Multicentre Prospective Longitudinal descriptive study	Patients with migraine and switching anti-CGRP mAbs treatment	49 patients: Erenumab to galcanezumab: 14 Galcanezumab to erenumab: 16 Erenumab to fremanezumab: 19				X	
Martínez (2021) (80) [ABSTRACT]	*Balearic Islands*	Single-centre Retrospective Longitudinal descriptive study	People with migraine treated with anti-CGRP mAbs	180 patients: Erenumab: 118 Galcanezumab: 56 Fremanezumab: 6			X	X	
Millán Vázquez (2021) (57) [ABSTRACT]	*Andalusia*	Multicentre Prospective Longitudinal and cohort descriptive study	Patients with migraine treated with anti-CGRP mAbs (erenumab and galcanezumab) with follow-up ≤6 months	399 patients: Erenumab: 270 Galcanezumab: 129	MHD	X		X	
Morollón-Sánchez-Mateos (2021) (81) [ABSTRACT]	*Catalonia*	Single-centre Retrospective Longitudinal descriptive study	CM non-responder patients (rMMD<50%) after 12 weeks of treatment	19 patients: Erenumab to fremanezumab: 10 Erenumab to galcanezumab: 5 Galcanezumab to erenumab: 4				X	
Paula Arias (2021) (58) [ABSTRACT]	*Catalonia*	Single-centre Prospective Longitudinal and cohort descriptive study	Patients with migraine treated with anti-CGRP mAbs	50 patients: Erenumab: 32 Galcanezumab: 9 Fremanezumab: 9	MMDs HIT-6				
Soler (2021) (82) [ABSTRACT]	*Balearic Islands*	Single-centre Retrospective Longitudinal and cohort descriptive study	Patients with migraine treated with anti-CGRP mAbs (erenumab and galcanezumab)	31 patients: Erenumab: 22 Galcanezumab: 9	MMDs Response rate	X			

ICHD-3, International Classification of Headache Disorders criteria; CGRP; calcitonin gene-related peptide; CM, chronic migraine; EM, episodic migraine; HFEM, High frequency episodic migraines; mAbs, monoclonal antibodies; MMDs, monthly migraine days; rMMDs, reduction of monthly migraine days; MHDs, monthly headache days; rMHD, reduction of monthly headache days; HIT-6, Headache Impact Test-6; MIDAS, Migraine Disability Assessment; PROs, patient-reported outcomes; VAS, Visual Analogue Scale; AMSMD, acute migraine-specific medication days; OnabotA; Onabotulinumtoxin A.

Most of the selected publications reported patients’ sociodemographic and clinical characteristics and clinical outcomes (*n* = 25, 86.2%). Thirteen (44.8%) of the publications included reported data on treatment persistence and adherence, while 18 (62.1%) reported on treatment patterns. Only five (17.2%) publications showed PRO data.

Most publications explored different anti-CGRPs treatments but reported disaggregated data on galcanezumab (*n* = 19, 65.5%), while others focused on galcanezumab only (*n* = 10, 34.5%).

The quality assessment showed that all (*n* = 6) selected articles met ≥54.6% of the STROBE recommendations (Supplementary Table S3).

### 3.2 Population characteristics

Twelve publications informed about gender of patients receiving galcanezumab, with a proportion of women ranging from 69 to 97%. In addition, 12 of the selected studies reported the mean age of patients treated with galcanezumab, which ranged from 43.6 to 56 years old, excluding one study conducted in patients older than 65 years of age (25).

Regarding patients’ clinical characteristics, 16 studies reported ratios of migraine types in patients treated with galcanezumab. Excluding the four studies restricted to patients with CM, the most frequent type of migraine was CM (61–88%), followed by EM (not better specified; 19–39%) and high frequency EM (7–12%).

On the other hand, information about the presence of medication overuse headache (MOH) was reported in six studies. Excluding the two studies in which MOH was an inclusion criterion, the prevalence of MOH ranged from 57 to 66%. In addition, 72.9% of patients experienced other concomitant condition (often anxiety or depression symptoms), and 46–49% may have fibromyalgia.

Baseline MMDs and monthly headache days was reported in nine and 11 studies, respectively. The baseline MMDs ranged from 12 to 24 (mostly 15–20), and the monthly headache days from 15 to 26 (mostly 20–25).

The subpopulation pre-treated with OnabotA was reported in eight studies, being 33.3 to 100.0% of the patients. Detailed data can be found in Supplementary Table S4.

### 3.3 Clinical outcomes

#### 3.3.1 Effectiveness and disease burden

Fifteen studies reported effectiveness and disease burden data on galcanezumab treatment. The effectiveness was mainly assessed by using MMDs (*n* = 7), monthly headache days (*n* = 8). And the disease burden by using the headache impact test-6 (HIT-6; *n* = 9), and migraine disability assessment (MIDAS; *n* = 4).

**Figure 2:**
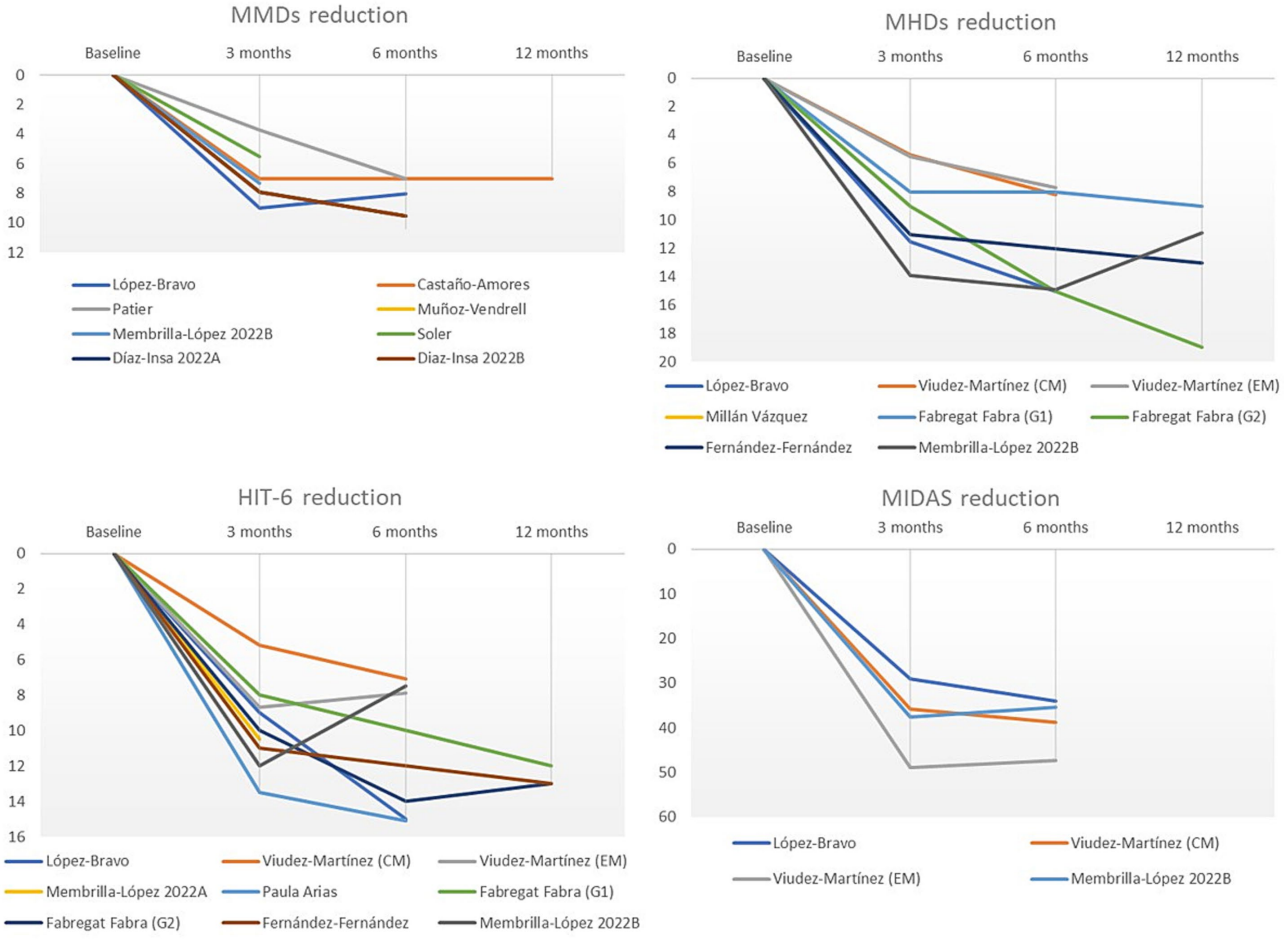
Graphical representation of galcanezumab effectiveness in patients with migraine evaluated using reduction of MMDs, monthly headache days, HIT-6 score, and MIDAS score.

Overall, all indicators showed a reduction within 3 months of treatment initiation, which was generally sustained after 6 and 12 months from baseline. The reduction in MMDs ranged from 3.7 to 9.0 after 3 months up to 7.0 to 9.5 after 6 months, compared to baseline and only one study reported the reduction at 12 months of 7.0. The reduction in monthly headache days ranged from 5.4 to 13.9 after 3 months, 7.7 to 15.0 after 6 months and up to 9.0 to 19.0 after 12 months, compared to baseline. Additionally, the range of reduction in scores of HIT-6 and MIDAS was 5.2 to 15.0 points and 29.0 to 48.9 points, respectively, from baseline (Figure 2; Supplementary Table S5). In those studies that compared differences from baseline, the decreases in MMD and monthly headache days were found to be significant (*p* < 0.005; Supplementary Table S5).

In addition, only the study conducted by Viudez-Martínez et al. (26) showed a reduction in pain intensity of −1.0 and −1.2 for CM and EM patients measured with a 10-point visual analogic scale, respectively, during the initial 6-month period measured using a visual analogue scale (VAS) from a baseline of 8.4 points in both CM and EM. The authors also reported a significant decrease in the number of days when acute migraine-specific medication was used, with both CM and EM patients experiencing a reduction of −8.1 and −4.9 days, respectively.

#### 3.3.2 Safety

A total of 12 studies reported adverse events (AEs) data (Supplementary Table S6). AEs associated with galcanezumab treatment were reported in treatment-naïve patients (not previously treated with an anti-CGRP; *n* = 9) and in patients who had switched from erenumab (*n* = 3; one study included both groups). Furthermore, four studies presented concomitant use of OnabotA.

The total frequency of AEs were reported in four studies, with an incidence of 1.3–37.5%. According to the type of AEs, the most commonly reported was constipation (7 out of 13 studies), ranging from 4 to 44%. Others such as injection site reactions, weight gain, wearing off effect, headache worsening, alopecia, dizziness, pruritus, diarrhoea, nausea, and toxicodermia were also reported (Supplementary Table S6).

### 3.4 Treatment persistence and discontinuation

**Figure 3:**
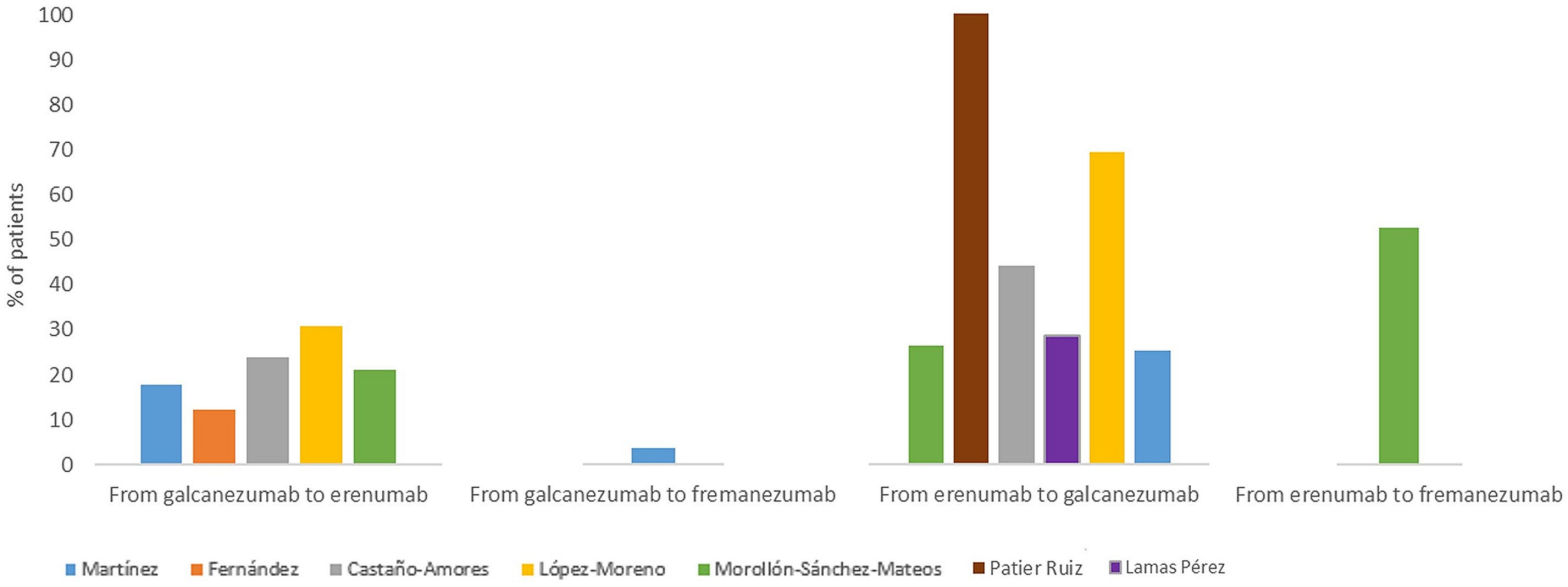
Galcanezumab persistence.

The persistence rate was assessed in five studies, varying between 94.4 and 95.7% at 3 months and 59.8 and 76.8% at 12 months of the study period (Figure 3).

Núñez-Lozano et al. (27) reported a persistence of 59.8% at 12 months in patients with migraine, and a history of multiple prior preventive treatment failures. The median duration of galcanezumab treatment reported was 182.9 (84–224) days, as reported by Patier-Ruiz et al. (28) and 14.6 (9.4–22.8) months, as reported by Núñez-Lozano et al. (27).

Twelve publications reported data on galcanezumab discontinuation, but only five provided specific information on the reasons for discontinuation (Supplementary Table S7). The most frequent reasons for discontinuation on the patients were ineffectiveness (ranging from 10.0 to 38.9%), improvement of the disease (ranging from 14.9 to 25.5%) and presence of AEs (<7% of the patients who discontinued galcanezumab treatment).

### 3.5 Treatment patterns

Most publications reported the duration of treatment for galcanezumab. Focusing on the number of doses of galcanezumab, the mean number of doses administered in different time periods varied from 3.0 at 3 months to 7.5 at 12 months (28–30).

**Figure 4:**
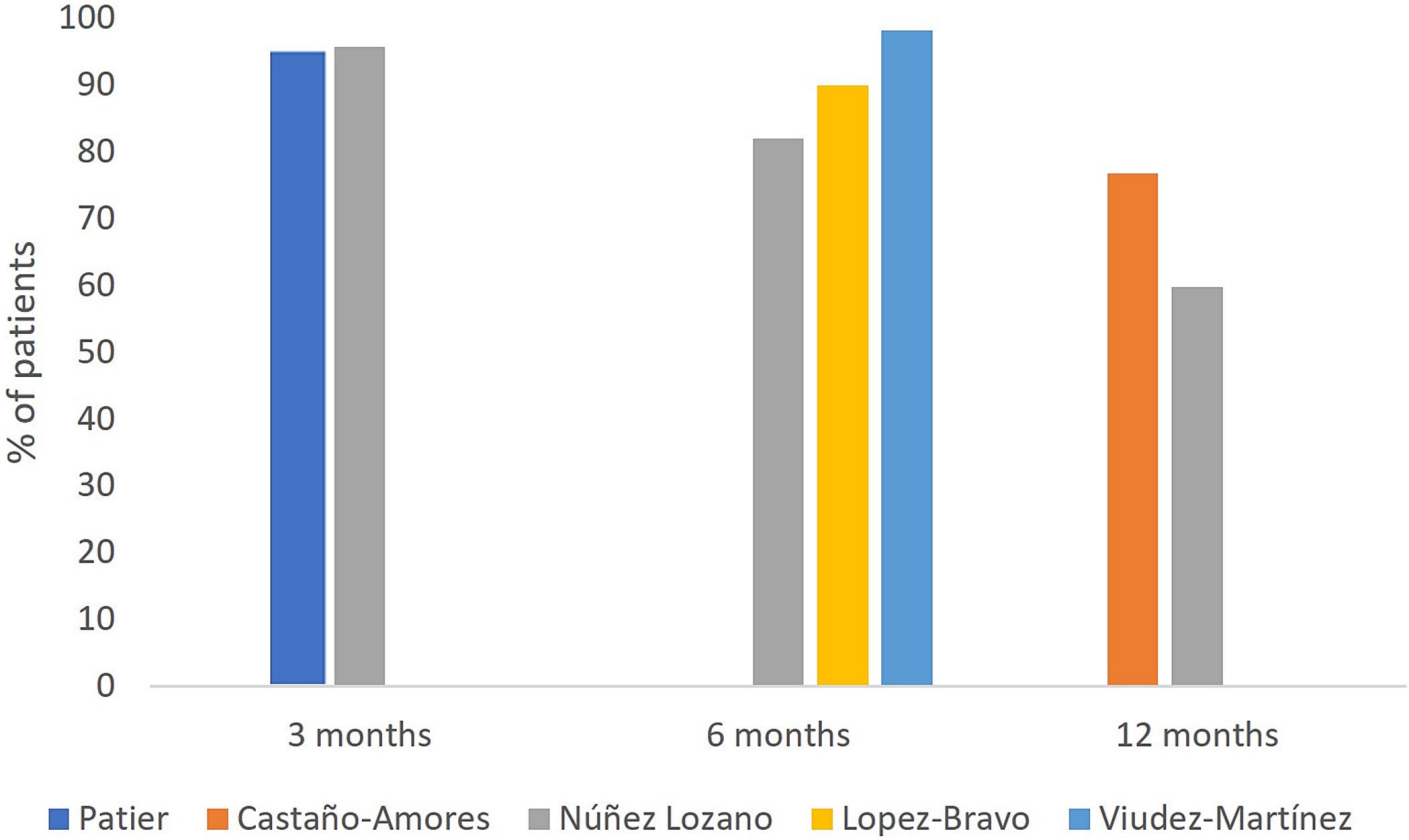
Summary of treatment switches.

Focusing on anti-CGRP treatments, seven studies reported treatment switching between mAbs, either from/to erenumab, galcanezumab and fremanezumab (Figure 4). Switching from erenumab to galcanezumab was reported in six studies applied on a design based on clinical practice (range 25.4–69.2% of patients), all of them reported switching due to inefficacy or AEs related to erenumab. On the other hand, switching from galcanezumab to erenumab was reported in five studies (range 17.8–30.8% of switching patients) and from galcanezumab to fremanezumab in 1 study (3.6% of switching patients; Figure 4).

**Figure 5:**
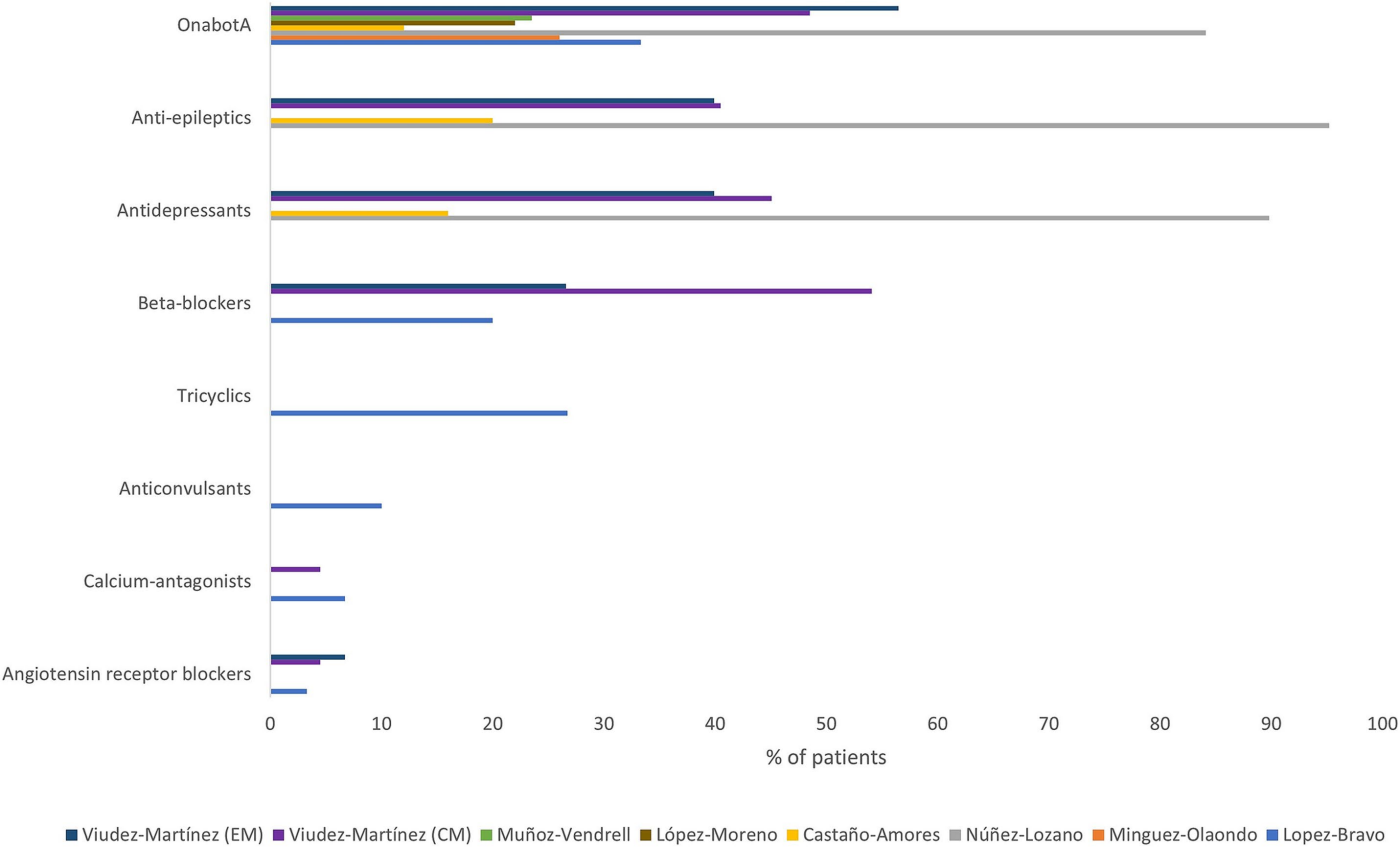
Concomitant preventive treatments. OnabotA, Onabotulinumtoxin A; CM, chronic migraine; EM, episodic migraine.

Concomitant treatments were reported in seven studies, being OnabotA, antidepressants and anti-epileptics the most commonly described (Figure 5). Concomitant medication refers to any medication taken simultaneously with galcanezumab treatment.

### 3.6 Patient-reported outcomes

Five publications included information regarding PROs (29, 31–34).

Lopez-Bravo et al. (31) assessed the subjective patient satisfaction with galcanezumab treatment using the self-reported Treatment Satisfaction Questionnaire for Medication (TSQM). This questionnaire scores from 0 to 100, with higher scores representing higher satisfaction in the domains. The median score at 12 and 24 weeks was significantly higher than the baseline for effectiveness, convenience, and global satisfaction (Table 3). Moreover, anxiety and depression symptoms were assessed using the self-administered Hospital Anxiety and Depression Scale (HADS-A and HADS-D). The level of anxiety of the patients at baseline was considered clinically significant since the score was ≥8 (Table 3). Both anxiety and depression scores significantly decreased after 12 weeks from the treatment initiation with galcanezumab, compared to baseline. The level of anxiety was further reduced after 24 weeks while the level of depression was sustained after 24 weeks and compared to 12 weeks (Table 3).

**Table 3:** Overall treatment satisfaction scores for each domain and HADS scores (Lopez-Bravo et al., 2022).

Score, median [IQR]	TSQM				HADS	
	Effectiveness	Side effects	Convenience	Global satisfaction	Anxiety	Depression
Baseline, *n* = 30	50.0 [44.4–50.0]	100.0 [76.6–100.0]	66.7 [61.1–83.3]	50.0 [30.4–50.0]	8.5 [6.2–12.7]	7.0 [4.5–10.0]
12 weeks, *n* = 30	80.6 [66.7–88.9], *p* < 0.001	100.0 [100.0–100.0], *p* = 0.010	83.3 [73.6–88.9], *p* = 0.001	78.6 [66.1–98.2], *p* < 0.001	6.5 [4.0–10.0], *p* = 0.021	3.0 [2.0–7.7], *p* = 0.011
24 weeks, *n* = 27	66.7 [66.7–83.3], *p* < 0.001	100.0 [100.0–100.0], *p* = 0.132	83.3 [80.6–97.2], *p* < 0.001	85.7 [64.3–92.9], *p* < 0.001	6.0 [2.5–9.0], *p* = 0.007	3.0 [1.0–7.5], *p* = 0.005

IQR, Interquartile range; TSQM, Treatment Satisfaction Questionnaire for Medication; HADS, Hospital Anxiety and Depression Scale.

In addition, four publications (29, 32–34) measured the subjective patient interpretation of symptom changes using the Patient Global Impressions scale (PGI). Castaño-Amores et al. (29) reported that 76% of patients using galcanezumab (19 out of 25) acknowledged a subjective improvement in intensity of migraine. Fernández Fernández et al. (33) informed that 91.7 and 89.6% of patients with galcanezumab treatment reported a global improvement after 3 and 6 months of treatment, respectively. In line, Fabregat-Fabra et al. (32) reported that patients felt better or much better after 12 months of treatment with galcanezumab, regardless of MMDs at baseline, with an improvement of 64.3% (30 MMDs) and 77.9% (<30MMDs). Interestingly, Minguez-Olaondo et al. (34) reported that patients combining galcanezumab treatment with OnabotA showed a worse global impression of illness management as compared to those patients without concomitant OnabotA.

No relevant data were found on health-related quality of life assessed by validated generic or specific questionnaires.

## 4 Discussion

In the present study, we systematically and comprehensively reviewed the existing real-world evidence on the use of galcanezumab in Spain. Since 2020, a total of 29 publications were identified encompassing a population of 2,592 Spanish patients with migraine. Notably, studies from the Galca-Only consortium are contributing to the understanding of the use of galcanezumab in our country as more than one third of these patients were enrolled in this study.

Our review shows that in Spain, patients treated with galcanezumab are mostly women with a range of a mean age of 43.6–56.0 years and who present mostly CM, which reflect the sociodemographic characteristics previously reported for the worldwide population with migraine, except for the higher prevalence of CM, reflective of Spanish uptake of this treatment, starting in more severely affected patients (2). The studies frequently described MOH, reported in up to 66% of patients when it was not an inclusion criterion. This is in line with migraine being described as the most common risk factor associated with MOH, affecting 78% of patients (35). Fortunately, recent studies have shown that the use of anti-CGRP mAbs was effective in this subpopulation (36–38).

In the reviewed studies, patients treated with galcanezumab showed a reduction in MMDs, monthly headache days, HIT-6 score and MIDAS score as early as 3 months after treatment initiation. These improvements were generally maintained or further enhance in responsive patients at 6 and 12 months. These findings indicate that the outcomes of galcanezumab in Spanish clinical practice are consistent with those observed in clinical trials and align with the results reported in previous real-world studies conducted in other countries (39–45). This demonstrates the effectiveness of galcanezumab in a variety of patients with CM and EM.

In an international context, it is noteworthy to mention the multicentre prospective cohort GARLIT study, which has reported on the galcanezumab use and its effectiveness in a real-life setting among Italian patients (46–51). While some studies reported the percentage of responders reducing 30% and/or 50% of monthly headache days, it remains challenging to draw definitive conclusions due to variations in patient populations and differing cut-off points (32, 33, 52–58). Summarising the results within a specific range or in a concise manner is particularly difficult.

Nonetheless, recent evidence suggests the need for a paradigm shift in the use of CGRP antagonists, such as galcanezumab, from being last-line treatments to becoming primary options in migraine management. The largest real-world study to date (59) found that patients with fewer migraine days and lower disability at baseline are more likely to respond well to treatment. This underscores the importance of initiating anti-CGRP therapy earlier to improve patient outcomes.

Regarding galcanezumab safety, it was suggested as a well-tolerated therapeutic option for patients with migraine since no serious AEs were reported. Treatment discontinuation due to inadequate tolerability occurred in <7% of cases (60). In our review, constipation was the most reported AE with heterogeneous prevalence ranging from 4 to 44%. Constipation frequency was also heterogeneous in previously published real-world studies; while is commonly reported as the most frequent AEs in some of these studies including all CGRP antagonists (39, 40, 48, 61), the majority of the studies reported injection site reaction as the most common AEs, with a frequency ranging from 8 to 34.6% (42–44). Other mild AEs such as dizziness, fatigue (42, 46, 60, 61) and nausea or vomiting (39) were reported. In general, these events occurred mainly in the first months and then tended to be resolved.

Treatment persistence rate observed in the reviewed studies was up to 59.8% at 12 months, a bit lower than the recent evidence published in Spain that is up to 71.4% (60). Although conclusions should be drawn with caution due to the mandatory discontinuation at 12 months in Spain that could influence this result and the same in other studies. Discontinuation was reported around 20–30%, and mostly due to ineffectiveness or improvement of the disease. Regarding the treatment discontinuation due to the positive disease progression, some studies showed that galcanezumab treatment effect was reduced during the post-treatment suspension period, decreasing over time, but not returning to baseline, in addition to patients not experiencing unexpected AEs (48, 62, 63). This information may be useful when treatment is stopped for a variety of reasons. Discontinuation due to AEs was low, revealing the good tolerability of galcanezumab. Data on discontinuation rates in other countries in the literature are scarce and heterogeneous and vary from approximately 3% (42, 64) at 3 months to 50, 43, and 32% at 6, 9 and 12 months, respectively (64).

In our systematic review, the maximum treatment duration reported was of 1 year, as it was the maximum follow-up time (28). However, it was suggested that patients with a higher baseline MMDs could be considered suitable candidates for continuing treatment for longer (28), which would allow the consolidation of the improvement before discontinuation (17). In line, Núñez-Lozano et al. (27) reported that galcanezumab demonstrated a high level of persistence in patients with severe migraine, particularly in those with CM and a history of multiple prior preventive treatments up to 12 months. Although clinical trials report similar efficacy between the different mAbs available, switching in clinical practice is recorded. Switching between erenumab and galcanezumab appeared to be slightly more frequent, although this may be due to the fact that they were marketed first in Spain, and therefore most data refer to this change in mechanism. Moreover, switching between mAb anti-CGRP in selected patients may be an option, although more studies would be required to establish the effectiveness of switching these treatments (65, 66).

Regarding concomitant treatments, OnabotA is the most frequently reported treatment, in line with previously published studies (67). A synergistic benefit of the two treatments is displayed, although evidence does not clearly state whether the concomitant preventive treatment is slowly titrated out or whether they are regular migraine therapies (67). The EHF (17) and SEN (68) guidelines recommend considering combination therapy for each individual situation.

Finally, Spanish patients reported increased global satisfaction with galcanezumab as well as decreased levels of anxiety and depression symptoms at 12 and 24 weeks after treatment initiation (31). These results are similar to those reported by Guerzoni et al., observing that HADS-A and HADS-D scores were significantly reduced in Italian patients with CM treated with galcanezumab (48). This might be due to the effect of galcanezumab on migraine relief, as has already been suggested by these authors, and the enhancement of the overall well-being and quality of life in individuals with migraines (69).

However, none of the studies in this review report health related quality of life using either generic or disease-specific questionnaires, such as the MSQ (Migraine Specific Quality of Life) questionnaire. While the exact cause remains unclear, this could be due to several factors, including the limited routine use of these measures in clinical practice or the lack of full integration of these assessments in observational research studies. Another possibility, as suggested by previous studies (70), is that current scales do not fully capture the multifaceted experience of living with migraine. Tools such as MIDAS and HIT-6, among others, has its advantages, but also significant limitations. These scores are often used as benchmarks for headache frequency or severity but do not consider other important aspects, such as the inter-ictal periods during which patients may still experience the broader impacts of the disease. This makes it challenging to accurately measure the full burden of migraine, especially given the irregular and variable nature of the condition. Therefore, it would be helpful to develop more comprehensive tools to better assess migraine-related disability, as well as to emphasise the collection of disease-specific health related quality of life data in future studies. Our review presents some limitations. First, a large number of conference abstracts were reviewed, and the information provided in them was reduced. In addition, most included studies were single-centre representing specific Spanish regions. However, considering that most of the Spanish regions were covered and multicentre national studies were also reviewed, the results may reflect the current situation of Spanish patients with migraine treated with galcanezumab. Additionally, although there are a few studies assessing the effectiveness of galcanezumab against other mAb anti-CGRP, these studies have a descriptive design, and therefore the comparative data are still missing.

In conclusion, this systematic literature review of real-world studies in Spanish patients with migraine treated with galcanezumab highlights the effectiveness of this treatment while being a well-tolerated therapeutic option.

## Data Availability

The original contributions presented in the study are included in the article/Supplementary material, further inquiries can be directed to the corresponding author.
