# Supplementary material for: Real-world experience of galcanezumab in the prevention of migraine in Spain: a systematic literature review
Source: Front Neurol. 2024 Nov 21;15:1502475. doi: 10.3389/fneur.2024.1502475 (PMC11619139; doi:10.3389/fneur.2024.1502475)
Supplement: Supplementary file 1 [file Table_1.DOCX]

Supplementary Material

# Supplementary Tables

Table S1. Search strategy and terms used in the different international and national databases consulted

| **PubMed/Medline** |
| --- |
| (“Migraine Disorders” [MeSH] OR “Migraine Disorders” [tiab] OR Migraine [tiab]) AND (Galcanezumab [tiab] OR Emgality [tiab] OR “anti-CGRP monoclonal antibody” [tiab] OR “humanized monoclonal antibody” [tiab]) AND (“Observational studies as topic” [MeSH] OR “Observational study” [Publication Type] OR Observational [tiab] OR “Cohort studies” [MeSH] OR Cohort [tiab] OR “Real-world evidence” [tiab] OR RWE [tiab] OR “Real world” [tiab]) AND (Spain OR Spanish [tiab] OR Andalucia [tiab] OR Andalusia [tiab] OR Aragon [tiab] OR Asturias [tiab] OR Balear* [tiab] OR Canarias [tiab] OR Canary [tiab] OR Cantabria [tiab] OR Castilla* [tiab] OR Castile [tiab] OR Cataluña [tiab] OR Catalonya [tiab] OR Valencia* [tiab] OR Extremadura [tiab] OR Galicia [tiab] OR Madrid [tiab] OR Murcia [tiab] OR Navarra* [tiab] OR Vasco [tiab] OR Basque [tiab] OR Rioja [tiab]) |
| **Cochrane** |
| ("Migraine Disorders OR "Migraine"[tiab]) AND ("Galcanezumab"[tiab] OR "Emgality"[tiab] OR " anti-CGRP monoclonal antibody "[tiab] OR “humanized monoclonal antibody” [tiab]) AND ("Observational studies as topic" [tiab] OR "Observational studies"[tiab] OR "Observational"[tiab] OR "Cohort studies"[tiab] OR "Cohort"[tiab] OR "Real-world evidence"[tiab] OR "RWE"[tiab])) |
| **Medicina en Español (MEDES)** |
| ("Migraine Disorders OR migraña"[todos] OR "Migraine"[todos] AND "Galcanezumab"[todos] OR "Emgality"[todos] OR " anti-CGRP monoclonal antibody "[todos] OR “humanized monoclonal antibody” [todos] AND "Observational studies as topic" [todos] OR "Observational studies"[todos] OR "Observational"[todos] OR "Cohort studies"[todos] OR "Cohort"[todos] OR "Real-world evidence"[todos] OR "RWE"[todos]) |
| **Índice Bibliográfico Español en Ciencias de la Salud (IBECS)** |
| (Migraine Disorders OR Migraine OR migraña)[palabras] OR (cefalea)[palabras] AND (Galcanezumab OR Emgality OR anti-CGRP monoclonal antibody OR humanized monoclonal antibody)[palabras] AND (Observational studies as topic OR Observational study OR Observational OR Cohort studies OR Cohort OR Real-world evidence OR RWE)[palabras] |

Filters applied: Publication dates, 2020 (August) - 2023 (August)

Table S2. Eligibility criteria used in the systematic review

| **Study Characteristic** | **Inclusion criteria** | **Exclusion criteria** |
| --- | --- | --- |
| **Patient population** | - Adult patients (>18) | - Patients with other therapeutic indication not included in the Summary of Product Characteristics (e.g. patients with cluster headache) |
| **Intervention- Treatment** | - Galcanezumab | - NA |
| **Intervention- Comparison** | - All interventions, placebo or usual care are eligible comparators | - NA |
| **Outcomes** | *Patients’ demographic and clinical characteristics*   - Age - Gender - Type of migraine   *Clinical outcomes*   - Effectiveness - Safety   *Treatment persistence and adherence*   - Time to discontinuation and re-initiation - Mean and median time on treatment)   *Treatment patterns*   - Previous treatments for migraine (% anti-CGRP monoclonal antibody-naïve patients; % patients that switch from other therapies) - Current concomitant treatments   *Patient-reported outcomes (PROs)*   - Treatment satisfaction - Quality of life - Other potential patient reported outcomes   *Outcomes by subpopulations of interest (Patient pre- treated with botulinum toxin)* |  |
| **Study design** | - Observational studies | - Clinical trials - Narrative reviews - Systematic reviews - Meta-analysis - Economic evaluations - Editorial - Opinion articles - Letters to the editor |
| **Time Frame** | - 2020 (August) - 2023 (August) for articles - 2021 (August) - 2023 (August) for conference abstracts | - Articles prior to 2020 (August) - Conference abstracts prior to 2021 (August) |
| **Language** | - English - Spanish | - Non-English or non-Spanish |
| **Country** | - Studies conducted in Spain or multi-country studies including data from the Spanish population | - Studies conducted outside Spain, not including/reporting data from the Spanish population. |

NA: not applicable

Table S3. Quality assessment of the selected full-text publications using STROBE

| **N item** | **Lopez-Bravo et al. 2022** | **Castaño-Amores et al. 2022** | **López-Moreno et al. 2022** | **Viudez Martinez et al. 2022** | **Patier Ruiz et al. 2022** | **Muñoz-Vendrell et al. 2023** |
| --- | --- | --- | --- | --- | --- | --- |
| 1 | 1 | 1 | 0 | 1 | 1 | 1 |
| 2 | 1 | 1 | 1 | 1 | 1 | 1 |
| 3 | 1 | 1 | 0 | 1 | 1 | 1 |
| 4 | 1 | 1 | 1 | 1 | 1 | 1 |
| 5 | 1 | 1 | 0 | 1 | 1 | 1 |
| 6 | 1 | 1 | 1 | 1 | 1 | 1 |
| 7 | 1 | 1 | 1 | 1 | 1 | 1 |
| 8 | 1 | 0 | 1 | 1 | 1 | 0 |
| 9 | 1 | 0 | 0 | 0 | 0 | 0 |
| 10 | 0 | 0 | 0 | 1 | 0 | 1 |
| 11 | 1 | 1 | 1 | 1 | 1 | 1 |
| 12 | 1 | 1 | 0 | 1 | 0 | 1 |
| 13 | 0 | 0 | 1 | 1 | 1 | 1 |
| 14 | 1 | 1 | 1 | 1 | 1 | 1 |
| 15 | 1 | 1 | 1 | 1 | 1 | 1 |
| 16 | 1 | 1 | 1 | 1 | 1 | 1 |
| 17 | 0 | 0 | 0 | 1 | 0 | 0 |
| 18 | 1 | 1 | 0 | 1 | 1 | 1 |
| 19 | 1 | 1 | 1 | 1 | 0 | 1 |
| 20 | 1 | 1 | 1 | 1 | 0 | 1 |
| 21 | 1 | 1 | 0 | 0 | 0 | 1 |
| 22 | 1 | 1 | 0 | 0 | 1 | 1 |
| **Total** | **19** | **17** | **12** | **19** | **15** | **19** |

Table S4. Sociodemographic and clinical characteristics at baseline

| **Author (year)** | **Relevant inclusion criteria** | **Sex, female (%)** | **Age, years,**  **median [IQR]**  ***mean (SD)*** | **Time since diagnosis, years median [IQR]**  ***mean (SD)*** | **Type of migraine (%)** | | | **Years of CM**  **median [IQR]** | **MOH**  **(%)** | **MMDs median [IQR]**  ***mean (SD)*** | **MHDs median [IQR]**  ***mean (SD)*** | **OnabotA pre-treatment**  **(%)** | **Previous preventive treatment failures, median [IQR] / mean (SD)** |
| --- | --- | --- | --- | --- | --- | --- | --- | --- | --- | --- | --- | --- | --- |
|  |  |  |  |  | **CM** | **EM** | **HFEM** |  |  |  |  |  |  |
| Lopez-Bravo (2022) (24) | - | 80.0 | 46 [36.3-55.0] | 22.0 [16.5-30.5] | 76.7 | 23.3 | - | 6.5 [3.2-30.5] | 60.0 | 12.0 [8.5-15.0] | 22.5 [15.7-30.0] | 33.3 | 5 [4.0-7.0] |
| Patier (2022) (25) | *-* | 73.3 | *51.1 (10.8)* | NA | 73.3 | 26.7 | - | NA | NA | *23.9 (7.1)* | NA | 100 | 5.4 (1.2) |
| Fabregat Fabra (2022) (26) | CM | NA | 51.0 (NA) -  49.0 (NA) | NA | 100 | - | - | *8 (NA)* | NA | NA | NA | NA | 4 (NA) |
| Fernández Fernández (2021) (27) | CM | 87.6 | *48.0 (NA)* | NA | 100.0 | - | - | NA | NA | NA | 20 [6-30] | NA |  |
| Fernández Fernández (2022) (28) | - | NA | NA | NA | 76.4 | 23.6 | - | NA | NA | NA | 20 [NA] | NA |  |
| Fernández Soberón (2022) (29) | - | 69.0 | *56.0 (NA)* | NA | 65.4 | 34.6 | - | NA | NA | NA | NA | NA | 5 (NA) |
| Membrilla- López (2021) (30) | MOH | 85.2 | *49.4 (11.8)* | NA | 72.2 | NA | NA | NA | 100 | 20 [14-30] | NA | 100 |  |
| Membrilla-López (2022) A(20) | MOH | NA | NA | NA | NA | NA | NA | NA | 100 | NA | 20.9 [15-30] | NA |  |
| Membrilla-López (2022) B(21) |  |  |  |  |  |  |  |  |  |  |  |  |  |
| Mínguez-Olaondo (2022) (31) | - | 83.1 | NA | NA | NA | NA | NA | NA | NA | NA | NA |  |  |
| Núñez Lozano (2022) (32) | - | 85 | *46.3 (12.6)* | NA | 80.6 | 19.4 | - | NA | NA | NA | NA | 100 | ≥6 |
| Obach Baurier (2022) A(22) | - | 83.1 | *50.0 (12)* | NA | 67.6-80.9 | NA | NA | 4-9 | NA | NA | 20 [12-30] | NA |  |
| Obach Baurier (2022) B(23) |  |  |  |  |  |  |  |  |  |  |  |  |  |
| Castaño-Amores (2022) (35) | CM and HFEM | 76.0 | *45.0 (NA)* | NA | 88 | - | 12 | NA | NA | 12 [8-15] | NA | 64 |  |
| López-Moreno (2022) (36) | CM and HFEM | AD: 50.0 | AD: 46.5 [NA] | AD: 27.5 (NA) | AD: 92.8 | - | AD: 7.1 | NA | AD: 57.1 | *13 (6)-13.5 (6)^¥^* | 16 (8)-19.2 (7.7) *^¥^* | 100 | 5.3 (NA) |
| Muñoz-Vendrell (2023) (37) | ≥65 years | AD: 74.1 | *69.8 (4.4)* | AD: 18 [14–26.5] | 82.4 | NA | - | AD: 10 [NA] | 65.9 | *17.6 (7.2)* | *22.7 (7.1)* | AD:87.0 |  |
| Viudez-Martínez (2022) (38) | 18-65 years | CM: 97.0  EM: 91.3 | CM: *46.9 (11.8)*  EM: *43.6 (9.9)* | NA | 61 | 39 | - | NA | CM: *84.85^¥^*  EM: *69.57^¥^* |  | CM: *20.4 (7.7)*  EM: *14.7 (6.2)* | 100 |  |
| Diaz-Insa (2021) (39) | - | AD: 81.5 | AD: 47.4 | NA | AD: 89 | AD: 11 | - | NA | NA | NA | 20.2 [NA] | NA |  |
| Díaz-Insa (2022-A) (40) | - | AD: 81.6 | AD: 46.8 | NA | AD: 88.9 | AD: 11.1 | - | NA | AD: 69 | 19.3 [NA] | NA | NA |  |
| Diaz-Insa (2022-B) (41) | - | AD: 81.6 | AD:46.8 (NA) | NA | NA | NA | NA | NA | AD: 69 | 19.3 [NA] | NA | NA |  |
| Gracia Moya (2022) (42) | - | AD:80.3 | AD:*46.7 (11.4)* | NA | 80.2 | 19.8 | - | NA | NA | AD: *21 (7.4)* | NA | NA |  |
| Millán Vázquez (2021) (45) | - | 80.6 | NA | NA | 80.6 | NA | NA | NA | NA | NA | NA | NA | 5.8 (1.4) |
| Paula Arias (2021) (47) | - | NA | NA | NA | 100 | NA | NA | NA | NA | *17.3 (NA)* | NA | NA |  |

AD: aggregated data; CM: chronic migraine; EM: episodic migraine; HFEM: high frequency episodic migraine; MHDs: monthly headache days; MMDs: monthly migraine days; NA: not available; OnabotA: Onabotulinumtoxin A; MOH: medication overuse headache; SD: standard deviation; IQR: interquartile range.

Table S5. Studies evaluating galcanezumab effectiveness in patients with migraine

| **Author (year)** | **MMDs reduction,**  **median [IQR]**  ***mean (SD)*** | | | **MHDs reduction,**  **median [IQR]**  ***mean (SD)*** | | | **HIT-6 reduction,**  **median [IQR]**  ***mean (SD)*** | | | **MIDAS reduction,**  **median [IQR]**  ***mean (SD)*** | | |
| --- | --- | --- | --- | --- | --- | --- | --- | --- | --- | --- | --- | --- |
|  | **3 mo** | **6 mo** | **12 mo** | **3 mo** | **6 mo** | **12 mo** | **3 mo** | **6 mo** | **12 mo** | **3 mo** | **6 mo** | **12 mo** |
| Lopez-Bravo (2022) (24) | 9.0 | 8.0 | **-** | 11.5 | 15.0 | - | 9.0 [20.5- 4.2] | 15.0 [22.0- 4.5] | - | 29.0 [53.2- 7.0] | 34.0 [72.5.-14.5] | - |
| Patier (2022) (25) | *3.7*  *(5.3)* | *7*  *(5.9)* | - | NA | NA | NA | NA | NA | NA | NA | NA | NA |
| Fabregat Fabra (2022) (26) | NA | NA | NA | *8-9* | *8-15* | *9-19* | *8-10* | *10-14* | *12-13* | NA | NA | NA |
| Fernández Fernández (2022) (28) | NA | NA | NA | *11* | *12* | *13* | *11* | *12* | *13* | NA | NA | NA |
| Membrilla-López (2021) (30) | *7.3* | - | - | NA | NA | NA | *10.5* | - | - | *37* | - | - |
| Membrilla-López (2022) A(20) | NA | NA | NA | *12.9- 13.9* | *13.4-14.9* | *10.9^-^* | *12* | *7.5* | - | *37.5* | *35.5* | *-* |
| Membrilla-López (2022) B(21) |  |  |  |  |  |  |  |  |  |  |  |  |
| Vargas Mendoza (2022) (33) | NA | NA | NA | NA | NA | NA | 13 | - | - | NA | NA | NA |
| Diaz-Insa (2022-B) (41) | *7.9* | *9.5* | NA | NA | NA | NA | NA | NA | NA | NA | NA | NA |
| Castaño-Amores (2022) (35) | *7* | *7* | *7* | NA | NA | NA | NA | NA | NA | NA | NA | NA |
| López-Moreno (2022) (32) | 3 (8.7) |  |  | 4 (3.4) |  |  |  |  |  |  |  |  |
| Millán Vázquez (2021) (45) | NA | NA | NA | - | *8.6 (8.8)* | - | NA | NA | NA | NA | NA | NA |
| Paula Arias (2021) (47) | NA | NA | NA | NA | NA | NA | 13.5 | 15.1 | - | NA | NA | NA |
| Soler (2021) (48) | 5.5 [8.6–0.8] | - | - | NA | NA | NA | NA | NA | NA | NA | NA | NA |
| Viudez-Martínez (2022) (38) | NA | NA | NA | **CM** 5.4  **EM** 5.5 | **CM** 8.2  **EM** 7.7 | - | **CM** 5.2  **EM** 8.7 | **CM** 7.1  **EM** 7.9 | - | **CM** 35.8  **EM** 48.9 | **CM** 38.7  **EM** 47.3 | - |

CM: chronic migraine; EM: episodic migraine; HIT-6: Headache Impact Test-6; MHDs: monthly headache days; MIDAS: Migraine Disability Assessment; MMDs: monthly migraine days; NA: not available; OnabotA: Onabotulinumtoxin A; SD: standard deviation

Orange, estimated in the present review from baseline differences. Membrilla-López (2022), and Membrilla-López (2022) are different publications pertaining to the same study.

Table S6. Reported adverse events

| **Author (year)** | **Previous CGRP mAb** | **Adverse event (%)** | | | | | | | | | | | |
| --- | --- | --- | --- | --- | --- | --- | --- | --- | --- | --- | --- | --- | --- |
|  |  | **Total** | **Injection site** | **Constipation** | **Weight gain** | **Wearing off effect** | **Headache worsening** | **Alopecia** | **Dizziness** | **Pruritus** | **Diarrhoea** | **Nausea** | **Toxicodermia** |
| Lopez-Bravo (2022) (24) | Naïve | NA | 6.7 | 6.7 | 6.7 | - | - | - | - | - | - | - | - |
| Patier (2022) (25) | Erenumab | NA | - | 6.6 | - | - | - | - | - | - | - | - | - |
| Fernández Fernández (2022) (28) | Naïve | **18.5** | - | - | - | - | - | - | - | - | - | - | - |
| Fernández Soberón (2022) (29) | Naïve | **21** | - | - | - | - | - | - | - | - | - | - | - |
| Diaz-Insa (2021) (39) | Naïve | NA | - | 25 | - | - | - | - | - | - | - | - | - |
| Castaño-Amores (2022) (35) | Naïve: 66%  Erenumab: 44% | NA | - | 4 | - | 12 | 4 | - | - | - | - | - | - |
| Gracia Moya (2022) (42) | Naïve | **1.27** | - | - | - | - | - | - | - | - | - | - | - |
| López-Moreno (2022) (36) | Erenumab | 55.5 | - | 44 | - | - | - | 11 | - | - | - | - | - |
| Muñoz-Vendrell (2023) (37) | Naïve | **27.1** | - | - | - | - | - | - | - | - | - | - | - |
| Soler (2021) (48) | Naïve | NA | 22.2 | - | - | - | - | - | - | - | - | - | - |
| Viudez-Martínez (2022) (38) | Naïve | **37.5** | - | 14.3 | - | - | - | - | 5.4 | 3.6 | 3.6 | 5.4 | 3.6 |

Table S7. Main reasons for discontinuation of galcanezumab treatment

| **Author (year)** | **Previous CGRP mAb** | **Concomitant treatments** | **Discontinuation** | | | |
| --- | --- | --- | --- | --- | --- | --- |
|  |  |  | **Total patients from the study** | **% of patients who discontinuated galcanezumab of the total sample due to the following specific reasons** | | |
|  |  |  |  | **Ineffectiveness** | **Adverse events** | **Improvement of the disease** |
| Lopez-Bravo (2022) (24) | Naïve | OnabotA  Anticonvulsants  Tricyclics  Angiotensin receptor blockers  Beta-blockers  Calcium-antagonists | 30 | 10.0 | - | - |
| Patier (2022) (25) | Erenumab | NA | 30 | - | Constipation: 6.6* | - |
| Fabregat Fabra (2022) (26) | Naïve | NA | 1004 | 24.2 - 38.9 | - | - |
| Fernández Fernández (2022) (28) | Naïve | NA | 73 | 13.96  *(48% of the total who discontinued [29.1%])* | 5.38  *(18.5% of the total [29.1%])* | - |
| Fernández Soberón (2022) (29) | Naïve | NA | 52 | 19.16  *(83.3% of the total who discontinued [23%])* | - | - |
| Membrilla-López (2022B) (21) | Naïve | NA | 46 | 6.8* | - | - |
| Núñez Lozano (2022) (32) | Naïve | NA | 314 | 27.70  *(57.6% of the total who discontinued [48.1%])* | - | 14.96  *(31.1% of the total who discontinued [48.1%])* |
| Obach Baurier (2022) A(22) | Naïve | OnabotA | 1004 | - | - | 19.6 - 25.5* |
| Obach Baurier (2022) B(23) |  |  |  |  |  |  |
| Castaño-Amores (2022) (35) | Naïve: 66%  Erenumab: 44% | OnabotA  Antidepressants  Anti-epileptics | 25 | 24* |  |  |
| Gracia Moya (2022) (42) | Naïve | - | 157 |  | 1.27 *(16.6 % of the total who discontinued [3.54%])* |  |
| Martínez (2021) (44) | Erenumab | - | 56 | 14.3* | - | - |
| Viudez-Martínez (2022) (38) | Naïve | OnabotA  Antiepileptic  Beta-blockers  Antidepressants  Angiotensin II receptor antagonists  Calcium antagonists | 59 | - | Toxicodermia: 1.79* | - |

CGRP mAb: Calcitonin Gene-Related Peptide monoclonal antibodies; OnabotA: Onabotulinumtoxin A
